# Proposed Core Competencies and Empirical Validation Procedure in Competency Modeling: Confirmation and Classification

**DOI:** 10.3389/fpsyg.2016.00273

**Published:** 2016-03-08

**Authors:** Anna K. Baczyńska, Tomasz Rowiński, Natalia Cybis

**Affiliations:** ^1^Management Department, Kozminski UniversityWarsaw, Poland; ^2^Institute of Psychology, Cardinal Stefan Wyszynski University in WarsawWarsaw, Poland

**Keywords:** core competency model, empirical validation, employee classification

## Abstract

Competency models provide insight into key skills which are common to many positions in an organization. Moreover, there is a range of competencies that is used by many companies. Researchers have developed core competency terminology to underline their cross-organizational value. The article presents a theoretical model of core competencies consisting of two main higher-order competencies called performance and entrepreneurship. Each of them consists of three elements: the performance competency includes cooperation, organization of work and goal orientation, while entrepreneurship includes innovativeness, calculated risk-taking and pro-activeness. However, there is lack of empirical validation of competency concepts in organizations and this would seem crucial for obtaining reliable results from organizational research. We propose a two-step empirical validation procedure: (1) confirmation factor analysis, and (2) classification of employees. The sample consisted of 636 respondents (*M* = 44.5; *SD* = 15.1). Participants were administered a questionnaire developed for the study purpose. The reliability, measured by Cronbach’s alpha, ranged from 0.60 to 0.83 for six scales. Next, we tested the model using a confirmatory factor analysis. The two separate, single models of performance and entrepreneurial orientations fit quite well to the data, while a complex model based on the two single concepts needs further research. In the classification of employees based on the two higher order competencies we obtained four main groups of employees. Their profiles relate to those found in the literature, including so-called niche finders and top performers. Some proposal for organizations is discussed.

## Introduction

Competency-based management is a major strategic approach for HR management and organizational change (McClelland, 1973) and may be critical in gaining and upholding a strategic advantage (Mitrani et al., 1992; Campbell and Luchs, 1997; Davenport and Prusak, 1998; Nadler and Tushman, 1999). It has become a key tool for use in recruitment and development as well as for implementing organizational changes (Shippmann et al., 2000). McLagan (1980, p. 23) introduced the concept of competency models for human resources development, which she defined as “decision tools which describe the key capabilities required to perform a job.” Competency modeling ties the derivation of job specifications to an organization’s strategy, since employees can be viewed as an organization’s most important “intangible asset.” Competency modeling is a very widespread theme in both management literature and practice (Wernerfelt, 1984; Prahalad and Hamel, 1990; Rumelt, 1991; Peteraf, 1993; Collis and Montgomery, 1995; Teece et al., 1997; Porter, 1998; Eisenhardt and Sull, 2001; Hoopes et al., 2003; Ludwig and Pemberton, 2011; Kozlenkova et al., 2014). This approach has become a crucial aspect of describing employees in terms of organizational goals.

Firstly, the aim of the article is to present the core competency model. Starting from the proposal of Prahalad and Hamel (1990), we analyze the literature on entrepreneurship and management in order to define what is most important for today’s organizations in building a competitive advantage on the market. We then propose a competency model which suited the needs of a rapidly changing market, while at the same time taking into account ‘stability’ and high standards of task performance in employee profiles. This allows us to identify potential employees and create development plans which in the long term perspective are one of the factors of building a competitive advantage. The proposal includes the results of research in the field of entrepreneurial orientation (Ireland et al., 2001; Kuratko, 2009).

Secondly, we propose a two-stage empirical verification procedure for the competency model which can be used to verify the model and classify employees based on employee testing. The first stage is to verify the assumptions of the competency model within a confirmatory factor analysis (CFA, Byrne, 2013; Brown, 2015) or multi-group CFA (Marsh and Balla, 1994; Cheung and Rensvold, 2002; Byrne, 2008). This procedure allows the verification of the assumed dependencies between competencies, e.g., their independence or lower order competencies within classes of higher-order competency. The second stage is the classification of employees in the verified model with cluster analysis according to method proposed by Asendorpf et al. (2001). Cluster analysis is an exploratory method of analyzing data which makes it possible to identify groups among employees, while keeping to the principle of maximizing differences between groups and minimizing within-group differences. Each group is described by specific combination of standardized mean scores on factors used in the analysis and can be treated as a prototype.

Usually, competency is defined in terms of the “knowledge, skills, abilities and other characteristics (KSAOs) that are needed for effective performance in a job” (Campion et al., 2011, 226, see also Mansfield, 1996; Parry, 1996; Kochanski, 1997; Mirabile, 1997; Green, 1999; Lucia and Lepsinger, 1999; Shippmann et al., 2000; Rodriguez et al., 2002). However, Shippmann et al. (2000) stress the fact that these models are implemented in organizations without validation procedures. The assumptions and catalog of competencies may differ among organizations, but validation of the model is an indispensable stage for obtaining reliable information about employee potential. Types of behavior, which underpin the operationalization of competencies, have normative and desirable values. The same conclusion is drawn by Barrett and Depinet (1991). They point out that although McClelland and Boyatzis (1980) claim that competencies are better indicators of performance in the workplace than traditional intelligence tests, there are no empirical studies to support this claim. Laber and O’Connor (2000) also point to the lack of empirical research in testing the effectiveness of competency models. At the organizational level, Sparrow (1995) shows that most of the claimed profits of competency models for firms are based on case studies, where research methodology was not reported at all. Finally, the benefits obtained from using competency models are probably unknown (**Table 1**).

**Table 1:** Competency definitions and their origins.

Author	Definition of competencies
Boyatzis, 1982	“The underlying characteristics of a person which may include a motive, trait, skill, aspects of one‘s self-image or social role, or a body of knowledge which he or she uses” (p. 21).
Brophy and Kiely, 2002	“Skills, knowledge, behavior and attitudes required to perform a role effectively” (p. 167).
Parry, 1996	“A cluster of related knowledge, attitudes, and skills that: (1) affects a major part of one’s job, (2) correlates with performance on the job, and (3) can be improved via training and development” (p. 60).
Tett et al., 2000	“An identifiable aspect of prospective work behavior attributable to the individual” (p. 215).
Klemp, 1980	“The underlying characteristic of a person which results in effective and/or superior performance on the job” (p. 21).
Lawler, 1994	Argues that skills are the basic building blocks of competencies and often uses the words interchangeably.
Thompson and Cole, 1997	“Integrated sets of behavior which can be directed toward successful goal accomplishment” (p. 52).
Woodruffe, 1992	“The set of behavior patterns that the incumbent needs to bring to a position in order to perform his or her task and function with competence” (p. 17).
Klein, 1996	Refers to observable behavior that superior performers exhibit more consistently than average performers.
Spencer and Spencer, 1993	“Motives, traits, self-concepts, attitudes or values, content knowledge, or cognitive or behavioral skills – any individual characteristic that can be measured or counted reliably and that can be shown to differentiate significantly between superior and average performers, or between effective and ineffective performers” (p. 6).

Many competency models are very well conceived in terms of objectives and professional requirements (Boyatzis, 1982), but few have been empirically verified and their assumptions have not been tested (Baczyńska and Wekselberg, 2009). Thus some researchers and authors view competency modeling with some degree of skepticism. The validity of “competencies” as measurable constructs appears to be at the core of this controversy (Barrett and Depinet, 1991; Lawler, 1996; Barrett and Callahan, 1997; Pearlman, 1997). If, at the level of theoretical assumptions, the models do not undergo verification, then the employee classification devised on the basis of these assumptions cannot be a reliable source of knowledge about an organization.

Boyatzis (1982) wrote the first empirically based and fully researched book on competency model development. He proposed that a competency model of managers should have two important aspects: descriptions of competency types as well as a description of the competency levels expected in organizations. Among the proposed models commonly referred to, we can find different numbers of basic competencies, i.e., Wood and Payne (1998) indicate 12 important competencies for recruitment and selection, and Curtis and McKenzie (2002) define eight employability skills. Meanwhile, the meta-analysis conducted by Kurz et al. (2004) of 20 competency models, comprising a total of 112 key measurements, led to the selection of Eight Great Competencies, which included: (1) leading and deciding, (2) supporting and cooperating, (3) interacting and presenting, (4) analyzing and interpreting, (5) creating and conceptualizing, (6) organizing and executing, (7) adapting and coping, (8) enterprising and performing.

The best summary of the above analyses would appear to be the comparison made by *Competency* in 1996 (Armstrong, 1999), which presents models from 126 organizations. The authors list the competencies which appear in all the analyzed models, to a large degree corresponding with the above mentioned studies. These competencies are: (1) communication; (2) focus on achievement and results; (3) focus on customer satisfaction; (4) cooperation; (5) leadership; (6) planning and organization; (7) awareness of commerce and trade; (8) flexibility, adaptability; (9) stimulating development in others; (10) problem solving. In this way the researchers attempted to create a set of basic competencies, which could be used not only as **a basic** set for leaders, but for all employees in organizations.

Traditionally, entrepreneurial orientation refers to the general conscious, systemic processes taking place in a firm which have an entrepreneurial character (Covin and Slevin, 1991). According to Ginsberg and Guth (1990), the term and definition of entrepreneurial orientation appeared in the context of creating a holistic organizational strategy (e.g., Mintzberg, 1973). Mintzberg et al. (1976) have stressed that creating strategies is “important, in terms of the actions taken, the resources committed, or the precedents set,” entrepreneurial orientation represents the policies and practices which are the background for taking entrepreneurial decisions and actions.

Furthermore, we can find a relationship between entrepreneurship and the competitive advantage of organizations (Miller, 1983; Covin and Slevin, 1989; Venkatraman, 1989; Zahra, 1991; Lumpkin and Dess, 1996; Wiklund, 1998, 1999). Zahra and Covin (1995) have shown that organizations distinguished by a high entrepreneurial orientation have products in the premium segment, achieve greater profits than market competitors and develop faster, overtaking their rivals. Other researchers have underlined the positive impact of entrepreneurial orientation on (1) knowledge and information flows in an organization (Floyd and Wooldridge, 1999), (2) staff motivation (Altinay and Altinay, 2004), (3) growth of sales (Covin et al., 2006), (4) creation and application of new knowledge (Li et al., 2009), (5) profitability (Baker and Sinkula, 2009), and (6) increased work satisfaction (Wiklund and Shepherd, 2005). A lack of entrepreneurial activity in today’s global economy can be a recipe for failure, while flexibility, speed, innovation, and entrepreneurial leadership are the cornerstones (Kuratko, 2009).

The basic dimensions of entrepreneurial orientation can be determined using an integrated review of literature on strategy and entrepreneurship (Miller and Friesen, 1978; Miller, 1983; Venkatraman, 1989; Covin and Slevin, 1991). According to Miller (1983), the three dimensions of entrepreneurial orientation are innovativeness, risk-taking and pro-activeness. The entrepreneurial orientation dimensions indicated above usually exhibit a high mutual correlation, ranging from *r* = 0.39 to *r* = 0.75 (Stetz et al., 2000; Richard et al., 2004; Bhuian et al., 2005; Tan and Tan, 2005). It is also for this reason that the majority of studies combine the dimensions into one factor (Naman and Slevin, 1993; Covin et al., 1994; Lee et al., 2001; Wiklund and Shepherd, 2003; Walter et al., 2006).

Although Hornsby et al. (2009) postulated that entrepreneurial actions are very important for an organization and should be presented at every level of management, it appears that this factor is not sufficient to ensure the long-term existence of a company. Entrepreneurial orientation drives the competitive advantage of human capital, as it promotes searching for ways of out-performing rivals through proactive and creative actions. The process of industrial deconcentration as well as the rapid development of the customer service sector has forced firms to individualize their offer and to decentralize management based on network structures. In turn, this has caused entrepreneurial orientation in organizations to take on new significance, not only at the top-management or organizational level, but also from the staff perspective.

Numerous reports in the literature confirm that organizations which possess entrepreneurial orientation achieve the organization’s goals in financial and non-financial terms and that this dimension plays an important role in building the advantage of those organizations on the market (Miller, 1983; Covin and Slevin, 1989; Venkatraman, 1989; Zahra, 1991; Zahra and Covin, 1995; Lumpkin and Dess, 1996; Wiklund, 1998, 1999; Floyd and Wooldridge, 1999; Altinay and Altinay, 2004; Wiklund and Shepherd, 2005; Covin et al., 2006; Baker and Sinkula, 2009; Li et al., 2009). Prahalad and Hamel (1990) took a similar approach by looking at the core competency of an organization. They describe a core competency as the strategic strength of an organization and what makes it competitive. Authors discuss the role that core competencies play in the competitiveness of a corporation and believe that corporations should build upon a core of shared competencies. Prahalad and Hamel (1990, p. 82) define a core competence as “the collective learning in the organization, especially how to coordinate diverse production skills and integrate multiple streams of technologies.” From the resource-based view, sustained competitive advantage is seen as deriving from a firm’s internal resources if these can add value, are unique or rare, are difficult for competitors to imitate and are non-substitutable (Ellström, 1992; Cappelli and Crocker-Hefter, 1996; Foss and Knudsen, 1996). In our opinion the core competency model should stress the importance of entrepreneurial orientation and the fact that the managerial mind-set must become an opportunity-driven mind-set, where actions are never constrained by resources currently controlled (Morris et al., 2011). This strengthens the role of every employee, focusing on the performance orientation of all the members of an organization as a constructive asset. The virtue of the core competence approach is that it “recognizes the complex interaction of people, skills and technologies that drives firm performance and addresses the importance of learning and path dependency in its evolution” (Scarbrough, 1998, p. 229).

In the literature there have been many conceptions of the basic competency model (Boyatzis, 2008; Velde, 2009), although most work in competencies and competency modeling has focused on the individual. For the purposes of our study, we asked 12 subject-matter experts from the field of management (three top level managers; three human resources managers working in organizations, three coaches working in different organizations; three academic staff from the field of management) to participate in our research. During an 8-h session using the Angoff (1971) method to reach consensus, they were asked to choose competencies which: (1) apply to every type of organization; (2) were considered to be the most universal or important in every workplace, regardless of the type of position; (3) guarantee the high standard and efficiency of tasks performed. We made use of the data from the article in *Competency* (see Armstrong, 1999) which listed ten competencies most commonly desired by organizations: (1) communication; (2) focus on achievement and results; (3) focus on customer satisfaction; (4) cooperation; (5) leadership; (6) planning and organization; (7) awareness of commerce and trade; (8) flexibility, adaptability; (9) stimulating development in others; (10) problem solving. During the session experts chose most essential and universal competencies in all types of organizations. The final proposal includes two orthogonal higher-order competencies, each consisting of three aspects (competencies) namely: higher-order performance competency or performance orientation and higher-order entrepreneurial competency or entrepreneurial orientation.

### Performance Orientation

**FIGURE 1:**
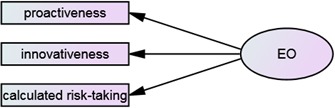
**Diagram of the entrepreneurial orientation (EO) and its aspects**.

**Figure 1** presents model of performance orientation, which consists of: (1) organization of work, i.e., the ability to order and prioritize the execution of tasks. It is manifested by the integration of individual actions for the timely execution of complex plans. It also means ensuring the efficient organization of work using available resources and possibilities; (2) cooperation, i.e., the ability to work effectively in a team and the willingness to help and support co-workers both in one’s own department and in the organization as a whole. It is expressed in nurturing good interpersonal relations and effective communication with others; (3) goal orientation, i.e., the readiness to focus on important and long-term objectives. Moreover, it means favoring lines of action which ensure high standards of work or tasks performed. This competency is linked with striving to maximize achievements and results, while at the same time coping with difficulties which occur at work.

### Entrepreneurial Orientation

**FIGURE 2:**
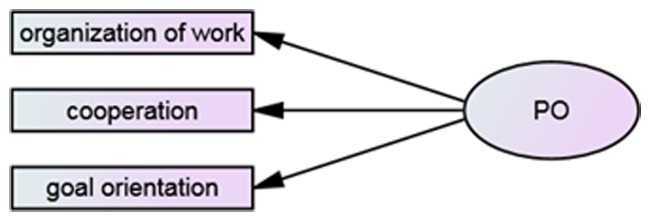
**Diagram of the performance orientation (PO) and its aspects**.

The second model, shown in **Figure 2**, describes enterprenuerial orientation consisting of: (4) pro-activeness, i.e., the initiative, energy and activeness which are needed to realize various career opportunities. It is a positive attitude toward diverse projects and tasks. It is also defined by the ability to motivate oneself to performing them at a rapid pace. It indicates the ability to cope with a large number of tasks to be executed within a similar timescale; (5) innovativeness, i.e., the ability to seek out and support new ideas. It is a desire to engage in creative processes which can lead to new products, services or processes. It also involves seeking information from different sources as well as the ability to connect them together in order to find new, practical solutions; (6) Calculated risk-taking, i.e., making good decisions in uncertain, risky situations. It is the ability to cope with uncertainty connected with the method of performing tasks. It is defined by the degree of efficiency of their execution with very little feedback. It also involves the ability to react appropriately to changing circumstances in order to minimize risk. The multi-dimensional nature of this competency corresponds to entrepreneurial orientation described by some other researchers (Burgelman, 1984; MacMillan and Day, 1987; Venkatraman, 1989; Hart, 1992).

There are two basic employee orientations in our model – higher-order performance competency and higher-order entrepreneurial competency. Based on these two dimensions we can explore the class of employees. It provides a strong empirical approach for identifying employee profiles. We assumed that in our model we can recreate the taxonomy of employee which were proposed by Koźmiński (2008). The first of these secures the competitive advantage of a firm (*niche finders*), while the second ensures the high quality of action and cooperation within an organization (*top performers*). Koźmiński (2008) highlighted the crucial role of niche finders in organizations but also stressed the importance of top performers, who maintain the quality of work, cooperation and achievement of goals established for building a competitive advantage.

Our research objective was to create a competency model based on two employee orientations and six competencies. In the first stage of statistical verification we attempted to verify the model based on two employee orientations and six competencies. In the next step of the procedure we described profiles differentiated at the staff level in organizations. Our competency model is based on two assumptions, which are given below:

$$$ Performance orientation is linked to those who ensure high standards of tasks performed on a daily basis in the workplace, i.e., organization of work, cooperation and goal orientation. We assume that a relatively high level of these competencies will characterize top performers (Koźmiński, 2008).

Competencies relating to entrepreneurial orientation, which are linked to building a market advantage, i.e., pro-activeness, innovativeness and calculated risk-taking – competencies conforming to the model presented in the studies by Miller (1983), among others. A relatively high level of the above competencies characterizes employees of the niche finders type (c.f. Koźmiński, 2008).

In order to test the model, we established the following hypotheses:

Performance orientation consists of three aspects: cooperation, organization of work and goal orientation.

Entrepreneurial orientation consists of three aspects: innovativeness, calculated risk-taking and pro-activeness.

Entrepreneurial and performance orientation are relatively independent of each other.

**FIGURE 3:**
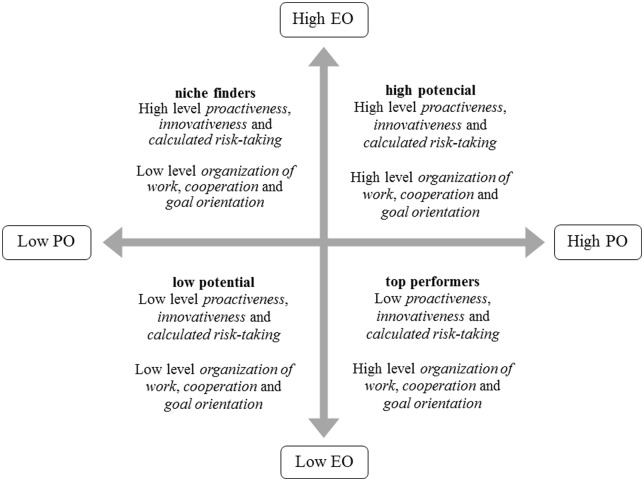
**A hypothetical model of employee classification based on the two independent dimensions – competencies relating to entrepreneurial orientation (EO) and competencies linked to performance orientation (PO)**.

Based on the two-dimensional model, as shown in **Figure 3**, employees can be classified into four groups: (1) high entrepreneurial orientation and high performance orientation (high potential); (2) high entrepreneurial orientation and low performance orientation (niche finders); (3) low entrepreneurial orientation and high performance orientation (top performers); (4) low entrepreneurial orientation and low performance orientation (low potential).

## Materials and Methods

In order to develop the measures, verify the competency model and established hypotheses we conducted two online studies (at the turn of the year 2014/2015), on Polish samples. In both studies, adult respondents were recruited by trained research assistants and given a password enabling them to fill in an on-line questionnaire. Participation was voluntary and anonymous. In line with ethical principles, participants were informed about the aim of the study and about the possibility of withdrawing from the research without any consequences at any time. The study did not require the consent of the ethical committee, because it did not contain any factors which might pose a risk to participants.

### Participants

We conducted the pilot study on a sample of 131 participants aged 18–79 years (*M* = 32.7; *SD* = 12.7; 7 respondents did not indicate their age). **Table 2** presents the characteristics of the pilot study sample.

**Table 2:** Pilot study: sample characteristics.

	*N*	Age	*M*	*SD*
Females	87	19–79	33.6	13.2
Males	44	18–61	30.1	11.6

The validation study sample consisted of 636 respondents aged 24–89 years (*M* = 44.5; *SD* = 15.1). The professional experience of participants ranged between 1 to 60 years (*M* = 20.1; *SD* = 13.7). More detailed characteristics of the validation study sample are presented in **Tables 3A,B**.

**Table 3a:** Validation study: sample characteristics.

	*N*	Age	*M*	*SD*
Females	316	24–88	44.1	14.9
Males	320	24–89	44.9	15.3

**Table 3b:** Validation study: professional experience of respondents.

	*N*	Years of work	*M*	*SD*
Females	316	1–60	19.4	13.1
Males	320	1–60	22.3	14.1

### Measure

For the measurement of professional competencies we constructed a self-report questionnaire, with questions reflecting modes of behavior that diagnose a given competency. The first step in creating the questionnaire was to devise a set of indicators for each competency, relating to the zone of reference in which a given competency would be demonstrated in a work situation. An example is the *decision* zone for the calculated risk-taking. Next, we devised survey questions based on these indicators – six for each competency (including three reverse coded), resulting in a pilot survey comprised of 36 questions. Respondents were asked to estimate to what degree they considered themselves to be similar to the person described. We used a 7-point Likert response scale, from 1 (*Completely dissimilar to me*) to 7 (*very similar to me*).

#### Pilot Study

We used the data obtained in the pilot study to test the psychometric properties of the questionnaire. Based on analyses of the discriminant validity of the questions and reliability, as well as an exploratory factor analysis, we modified over half of the items in order to improve their psychometric properties. The modified survey was then used in a further study. We maintained the same number of questions and response scale in the revised version of our measure.

#### Validation Study

As before, we analyzed the revised questionnaire for the reliability and discriminatory power of questions and as a result two items with the weakest psychometric properties were removed from each rating scale, reducing the final range of the scales to four items. The reliability indices (Cronbach’s α) obtained for the six survey scales ranged from α = 0.60 to α = 0.83, while the average reliability coefficient of the questionnaire was 0.70, which can be accepted as satisfactory. The reliability calculated for entrepreneurial orientation and performance orientation reached α = 0.89 and α = 0.78 respectively. **Table 4** details the reliability indices of the revised questionnaire.

**Table 4:** Reliability of the revised questionnaire (*N* = 636).

Scale	Cronbach’s α
Proactiveness	0.81
Innovativeness	0.83
Calculated risk-taking	0.63
Organization of work	0.68
Cooperation	0.68
Goal orientation	0.60
**Entrepreneurial orientation**	**0.89**
**Performance orientation**	**0.78**
**Average**	**0.70**

The average item discrimination coefficient, estimated as the item-total correlation, ranged from 0.39 (goal orientation scale) to 0.65 (innovativeness scale). For the entrepreneurial orientation scale the average discrimination coefficient reached 0.58, while for performance orientation it reached 0.43. Item discrimination coefficient ranges for particular scales are shown in **Table 5**.

**Table 5:** Discrimination coefficients of the items in the revised questionnaire (*N* = 636).

Scale	Coefficient value range	Average coefficient
Proactiveness	0.61–0.65	0.63
Innovativeness	0.58–0.70	0.65
Calculated risk-taking	0.35–0.47	0.42
Organization of work	0.40–0.55	0.48
Cooperation	0.40–0.57	0.48
Goal orientation	0.31–0.48	0.39
**Entrepreneurial orientation**	**0.37–0.70**	**0.58**
**Performance orientation**	**0.28–0.50**	**0.43**

We also examined the shape of the distribution in each scale. Skewness and kurtosis take values conforming to those anticipated in normal distribution, ranging between < -1; 1 >. **Table 6** presents descriptive statistics of the scales.

**Table 6:** Descriptive statistics of the revised questionnaire (*N* = 636).

			Skewness		Kurtosis	
Scale	*M*	*SD*	Statistic	*SD*	Statistic	*SD*
Proactiveness	4.27	1.34	–0.15	0.09	–0.55	0.19
Innovativeness	4.63	1.31	–0.52	0.09	–0.22	0.19
Calculated risk-taking	4.62	1.02	–0.16	0.09	–0.03	0.19
Organization of work	5.24	0.96	–0.64	0.09	0.22	0.19
Cooperation	5.45	1.01	–0.79	0.09	0.43	0.19
Goal orientation	4.80	0.92	–0.25	0.09	0.18	0.19
**Entrepreneurial orientation**	**4.51**	**1.06**	**–0.27**	**0.09**	**0.03**	**0.19**
**Performance orientation**	**5.16**	**0.74**	**–0.41**	**0.09**	**0.64**	**0.19**

### Procedure

In order to test the established hypotheses, we carried out CFA and cluster analysis on the sample from the validation study (*N* = 636). Before proceeding with the cluster analysis, outliers were identified using boxplot. Observations outside 1.5 times the interquartile range above the upper quartile and below the lower quartile were treated as outliers and excluded from the analyzed sample, as their influence could distort the picture of observed clusters. Following this procedure, a sample of 619 respondents was obtained and used for analysis. We performed the CFA in order to test hypotheses H1–H3. Two indices were used in order to estimate how our model fit the empirical data: the *comparative fit index* (CFI) and the *root mean square error of approximation* (RMSEA). Some researchers recommend RMSEA indices below 0.06 or 0.08 (Bentler, 1990; Browne and Cudeck, 1992; Hu and Bentler, 1999). In line with the recommendation of Hu and Bentler (1999) we assumed that the model is acceptable (good fit with data), when CFI > 0.90; RMSEA < 0.08.

Hypothesis H4 was tested in a classification procedure using cluster analysis, according to the method proposed by Asendorpf et al. (2001). This method combines Ward’s hierarchical classification method with the non-hierarchical *k*-means method, in a cross-validation procedure performed on randomly divided halves of the study sample. After conducting the analysis, the agreement of the identified types (clusters) in both halves was calculated. In our study, we estimated the split-half agreement using Cohen’s kappa coefficient, which gives a value in the range of < -1, 1>, where 1 indicates complete agreement, and 0 indicates random distribution. The analysis was performed on the two variables: entrepreneurial orientation and performance orientation, which were standardized beforehand.

## Results

### Verification of the Competency Model

**FIGURE 4:**
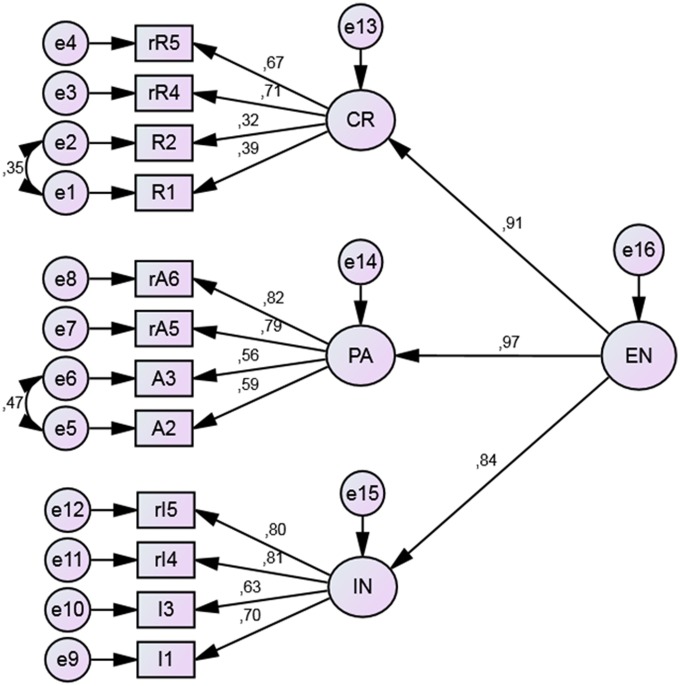
**Results of the confirmatory factor analysis (CFA) for the Entrepreneurial Orientation model.** EN, entrepreneurial orientation; CR, calculated risk-taking; PA, pro-activeness; I, innovativeness. Rectangular boxes represent observed variables (items). Factor loadings are placed over the arrows. The letter *r* marks reversed questions.

The CFA results for the entrepreneurial orientation model and its three dimensions: calculated risk-raking, pro-activeness and innovativeness, as shown in **Figure 4**, were as follows: χ^2^ = 242.98; df = 49; CFI = 0.94; RMSEA = 0.08 (0.07 - 0.09). The model was thus positively verified.

**FIGURE 5:**
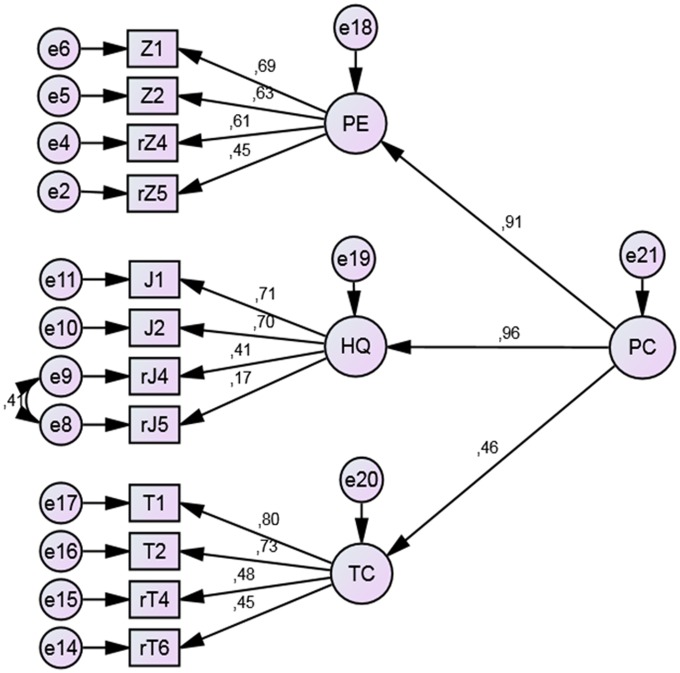
**Results of the CFA for the Performance Orientation model.** PC, performance orientation; PE, organization of work; HQ, goal orientation; TC, cooperation. Rectangular boxes represent observed variables (items). Factor loadings are placed over the arrows. The letter *r* marks reversed questions.

Next, we tested the confirmatory model of performance orientation incorporating its three aspects: organization of work, goal orientation and cooperation, as presented in **Figure 5**.

The fit indices obtained for the performance orientation model are satisfactory: χ2 = 195.05; df = 50; CFI = 0.92; RMSEA = 0.07 (0.06 - 0.08). We can therefore conclude that the performance orientation model fits well to empirical data.

**FIGURE 6:**
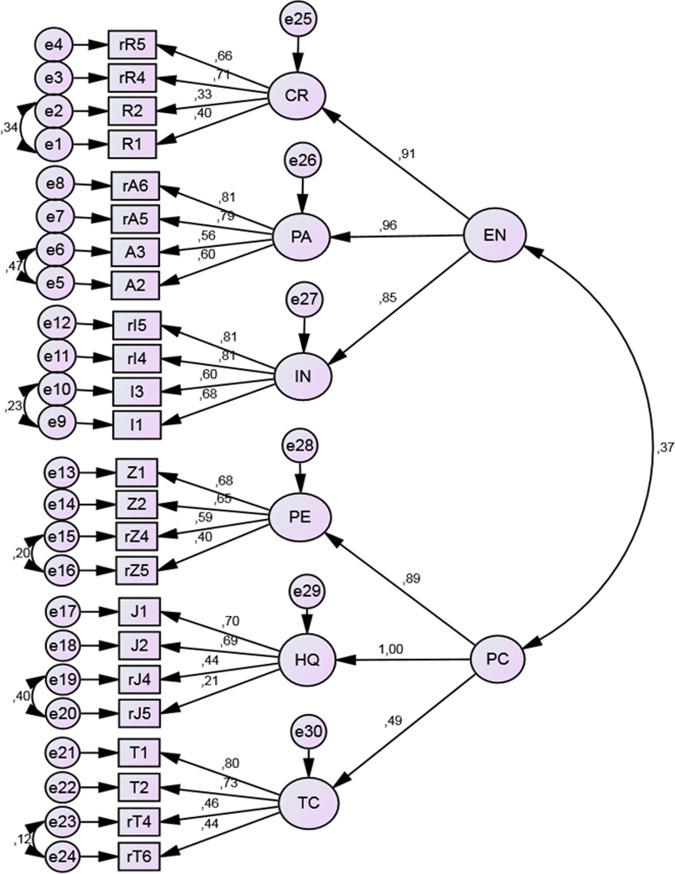
**Results of CFA of Entrepreneurial and Performance Orientation model.** PC, performance orientation; PE, organization of work; HQ, goal orientation; TC, cooperation. Rectangular boxes represent observed variables (items). Factor loadings are placed over the arrows. The letter *r* marks reversed questions.

**Figure 6** presents the tested model of entrepreneurial and performance orientation (hypothesis H3), along with factor loadings and correlation coefficients between the two dimensions. The fit indices for this model were as follows: χ2 = 1162.80; df = 239; CFI = 0.84; RMSEA = 0.08 (0.07 - 0.08). According to studies by Kenny and McCoach (2003) the CFI value of complex models tends to be lower even when the model is accurate, however, the observed decrease of CFI for this model is larger than expected and suggests a mediocre fit.

We assumed that both competencies would create one model, whilst maintaining a relative degree of independence between entrepreneurial and performance orientation. The correlation coefficient between the two dimensions is relatively low (*r* = 0.37) and the resulting common variance ratio for both orientations is 19.69%. This demonstrates their relative independence. However, complex model needs some improvements to meet expected criteria.

### Employee Taxonomy

**FIGURE 7:**
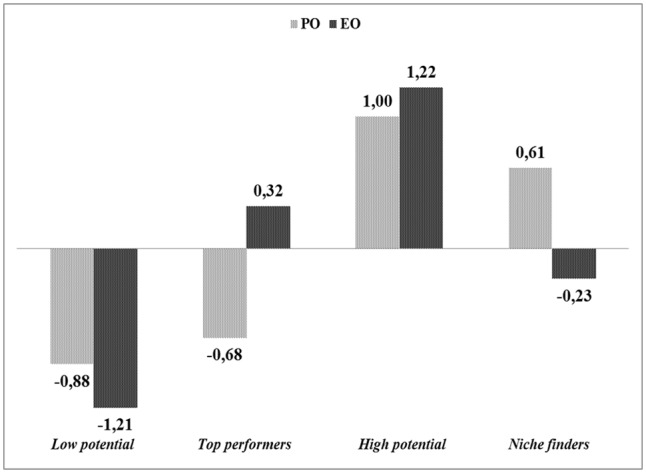
**Results of employee classification based on scores in entrepreneurial (EO) and performance (PO) orientation (***N*** = 619).** Values represent mean standardized scores on PO and EO dimensions for each cluster: Low potential employees (*N* = 153), top performers (*N* = 160), high potential employees (*N* = 141) and niche finders (*N* = 165).

According to the proposed model, four types of employees were identified. The mean Cohen’s kappa for the four identified clusters reached κ = 0.52 and can be interpreted as moderate, as it did not meet the suggested κ = 0.60 cut-off point indicating a good fit (De Fruyt et al., 2002; Asendorpf, 2003). **Figure 7** presents the four employee types identified through cluster analysis described by the standardized scores on the scales of entrepreneurial and performance orientation.

The first group identified were low potential employees (*N* = 153), with the lowest scores in both dimensions: entrepreneurial and performance orientation. The second group were top performers (*N* = 160), characterized by high scores in performance orientation, but with lower scores in entrepreneurial orientation. The third group comprised high potential employees (*N* = 141), characterized by high levels of both orientations. The last group identified were niche finders (*N* = 165), achieving high scores in entrepreneurial orientation but lower scores in performance orientation.

## Discussion

The concept of *competence* or *competency* is ubiquitous in today’s organizations and since the 1990s has dominated in the literature on strategic management (Mitrani et al., 1992; Campbell and Luchs, 1997; Nadler and Tushman, 1999). In this area particular emphasis is placed on *core competence* as the main source of ensuring the *competitive advantage of an organization* (Hamel and Prahalad, 1994).

In this article, we stress the fact that validation studies on the concept of competencies and the models on which organizations base their **employment policy** present a major problem. There is rarely any mention of methodical validation of the models which modern organizations rely on. This lack of empirical verification can lead to misguided decisions, based on unreliable data. In this article we try to fill this crucial gap. We present a two-step procedure for the validation of the competency model based on CFA (Byrne, 2013; Brown, 2015) and employee classification with cluster analysis (Asendorpf et al., 2001). We take this to be a starting point for devising a standard for competency models. With reference to studies by Thompson et al. (1996) and Scarbrough (1998), as well as Hamel and Prahalad (1994), we have proposed a model of core competencies which ensure the important needs of the modern organization. The advantage of the core competence approach is that it “recognizes the complex interaction of people, skills and technologies that drives firm performance and addresses the importance of learning and path dependency in its evolution” (Scarbrough, 1998, p. 229). Thompson et al. (1996) stress the fact that core competencies are similar in most organizations and their number is, and must be, limited.

The first objective of the article was to devise a model of core competencies. We started from the proposal of Prahalad and Hamel (1990) and defined what is most important for today’s organizations in building competitive advantage on the market. In line with this understanding of the concept, we proposed a core competency model which suits the needs of a rapidly changing market while at the same time taking into account “stability” and high standards of task performance in employee profiles. We proposed two dimensions that are important in the majority of organizations: (1) maintaining stability and high standards of performance in everyday tasks (performance orientation), (2) ensuring the organization’s competitive advantage on the market (entrepreneurial orientation).

In our verification of the model we confirmed the assumptions that: (1) performance orientation consists of three aspects: organization of work, cooperation and goal orientation, and that these are inter-related; (2) entrepreneurial orientation is comprised of three inter-dependent competencies: pro-activeness, innovativeness and calculated risk-taking. The third step shows that the complex model needs some further research. We believe that CFA is a useful tool for empirical verification which can be easily used by specialists in organizations.

Furthermore, using the verified model, we explored the taxonomy of employees with clustering procedure, based on two dimensions. Four types of employees – partially corresponding with Koźmiński’s (2008) typologies – niche finders, top performers, low and high potential, have direct and indirect impact on the fate of an organization. One kind is particularly desirable, i.e., high potential employees. Owing to the fact that the replicability of the four types falls slightly below the suggested κ = 0.60 cut-off point in *k*-means, our classification should be treated as a starting point for further empirical and statistical verifications, i.e., with three classes of employees. Moreover, our research proposes a taxonomy of employees that could be helpful, i.e., in recruitment and development processes. Organizations can also apply these findings in their long-term staff development plans. The classification of employees could be conducted using a different procedure, i.e., Latent Class Analysis (LCA, McCutcheon, 1987; Magidson and Vermunt, 2002).

From a practical point of view, the results above provide a good starting point for further research and discussion on the empirical verification of competency models used in organizations. It would be worth analyzing whether the employee profile translates into organizational efficiency; or whether the human potential of individual organizations described in terms of high potential, niche finders and top performers translates into high organizational performance and vice versa. Thereby, we can determine the number of employees who fit one of the four mentioned profiles in an organization, or in other words, we can determine the profile of staff potential in a given organization and attempt to shape it in a specified direction. We can define the employee profile for a given position or trade, or formulate character profiles for individual segments of the market. Systematic research in this area is required in order to enable better predictions of employee behavior in different contexts and situations.

### Limitations

The conducted study is not without its limitations. Firstly, it is important to verify our core competency model in different groups and cultures, i.e., not only among middle managers or specialists. This could show its universal use and would allow comparability in terms of work positions (so-called invariance) or types of culture in which a given company operates. Our research was limited to one cultural context, which may not be comparable with other possible contexts. The environmental (cultural) factor may be a moderator of individual dependencies. Secondly, it may be that linking organizational efficiency with a core competency model is not always justified. The assumption that entrepreneurial orientation is – directly or indirectly – linked to organization’s advantage needs to be empirically verified in further research.

## Author Contributions

The paper was prepared by three authors: AB, TR, NC. All authors take an active part in all stages of preparing process of this paper. Below are listed all stages of our work: Design of the work; preparing the new tool; the acquisition, analysis, and interpretation of data for the work; (AB, TR, NC). Drafting the work or revising it critically for important intellectual content; (AB, TR, NC). Final approval of the version to be published; (AB, TR, NC). Agreement to be accountable for all aspects of the work in ensuring that questions related to the accuracy or integrity of any part of the work are appropriately investigated and resolved (AB, TR, NC).

## Conflict of Interest Statement

The authors declare that the research was conducted in the absence of any commercial or financial relationships that could be construed as a potential conflict of interest.
